# Longitudinal patterns in objective physical activity and sedentary time in a multi‐ethnic sample of children from the UK

**DOI:** 10.1111/ijpo.12222

**Published:** 2017-05-22

**Authors:** L. Smith, D. Aggio, M. Hamer

**Affiliations:** ^1^ The Cambridge Centre for Sport and Exercise Sciences, Department of Life Sciences Anglia Ruskin University Cambridge UK; ^2^ UCL Department of Primary Care and Population Health, UCL Medical School, UK; UCL Physical Activity Research Group University College London London UK; ^3^ School of Sport, Exercise & Health Sciences Loughborough University

**Keywords:** diabetes, ethnicity, longitudinal, physical activity, sedentary

## Abstract

**Background:**

Children of South Asian decent born in the UK display lower levels of physical activity than British Caucasians although no longitudinal data are available.

**Objectives:**

We aimed to investigate change in activity levels over 1 year in a diverse ethnic sample of children residing in London, UK.

**Methods:**

Children were categorized into ethnic groups (Caucasian/mixed, Black, South Asian). At baseline and 1‐year follow‐up, children's objective physical activity was monitored (Actigraph accelerometer) for at least 1 day. Mixed models were employed to investigate differences in change in activity levels between ethnic groups.

**Results:**

A total of 281 children were included in the analyses. South Asians had a significantly greater increase in time spent sedentary at follow‐up than those of a Caucasian/mixed ethnicity (B [ratio sedentary/wear time] = 0.024; 95% confidence interval 0.003, 0.046). South Asian children recorded lower moderate to vigorous physical activity at baseline (B = −6.5, 95% confidence interval, −11.1, −1.9 min d^−1^, *p* = 0.006) although levels remained relatively stable over follow‐up and changes did not differ across ethnic group.

**Conclusions:**

In a diverse ethnic sample of children from inner city London, those of a South Asian ethnicity exhibited a significantly greater increase in sedentary time over a period of 12 months in comparison with Caucasian/mixed and Black children.

## Introduction

Diabetes prevalence is strongly associated with ethnicity. The Health Survey for England showed that all minority ethnic groups have a higher risk of diabetes compared with the general population 1. Compared with Caucasians, British South Asian adults are at an increased risk of type II diabetes 2, 3. South Asians show evidence of marked insulin resistance, hypertriglyceridemia and central adiposity 4. There is increasing evidence to suggest that differences in risk of type II diabetes emerge in early life. Indeed, British South Asians are at increased risk of type II diabetes in childhood and adolescence and show evidence of insulin resistance from 10 years of age 4, 5, 6.

A large body of literature exist that shows regular participation in physical activity aids in the prevention of both obesity and type II diabetes 7, 8. Moreover, a growing body of literature is emerging that suggests increasing sedentary time may also be contributing to the diabetes epidemic 9, 10, independently of physical activity. National survey data have suggested that a large proportion of children in the UK do not achieve current physical activity recommendations 11, 12, and this is particularly apparent in deprived inner city areas. Recent research, although limited, suggests that differences exist in children's activity levels between ethnic groups in the UK 13, 14. For example, compared with Caucasians 9–10 year olds, South Asians recorded 41 fewer accelerometer (Actigraph‐GT1M; an objective measure of free‐living activity) counts per minute and fewer minutes in moderate and vigorous physical activity (MVPA) and more time in sedentary activity 13. Another cross‐sectional study of primary school children 14 found that 73% of Caucasians and only 35% of South Asians achieved 60 min of MVPA daily. Both studies concluded that South Asian children participate in significantly less physical activity than Caucasians in the UK. Studies using subjective measures of physical activity have found similar findings 15. However, previous studies are all of a cross‐sectional design. Therefore, it is not known how activity levels in specific ethnic populations of children change over time. It has been suggested that physical activity decreases with age throughout childhood and adolescence 16; however, UK studies demonstrating this have used samples of predominantly Caucasian children, and differences in changes in activity levels have not been investigated between children of different ethnicity. One UK study 16 monitored objective physical activity at baseline and 1‐year follow‐up in a sample of 844 children (baseline 9–10 yeas; over 90% Caucasian). It was found that physical activity decreased over 1 year with 70.4% of children meeting physical activity recommendations at baseline and 65.8% at follow‐up (*p* < 0.001). Understanding how levels of activity change over time in different ethnic populations is essential for the development of targeted physical activity promotion and sedentary behaviour reduction interventions and thus aids in the prevention of obesity and type II diabetes.

The present study aims to investigate changes in activity levels over 1 year in a diverse ethnic sample (Caucasian, mixed, South Asian, Black) of inner city children residing in Camden, London, UK.

## Methods

### Study design

Camden Active Spaces was a school‐based quasi‐experimental study examining physical activity before (summer term 2014) and after (summer term 2015) playground renovation. Because no overall effects of the intervention was observed, we collapsed the sample and adjusted for intervention/control group in the present analysis. The study protocol has previously been published 17. Head teachers from each school provided explicit written consent for their school and school children to take part in the study. Parents were given the option to ‘opt‐out’ their child(ren). Ethical approval was granted by the University College London Research Ethics Committee (4400/002).

### Outcome measure: physical activity

Trained researchers fitted accelerometers (Actigraph GT3X) to children at baseline and follow‐up. Children were asked to wear the device around their waist during waking hours every day for seven consecutive days, but not during water‐based activities or sleep. Devices were programmed to sample at 30 Hz. Our protocol followed methods used in the International children's Accelerometery Database study 18. Data files were re‐integrated to a 60‐s epoch, and non‐wear time was defined as 60 min of consecutive zeros, allowing for 2 min of none zero interruptions. The first partial day of wear was excluded from our analyses in order to reduce the possibility of reactivity to wearing the device. All children with at least one school day and at least 500 min of measured monitor wear time between 07:00 AM and midnight were included. Time spent sedentary was defined as all minutes less than 100 cpm, light activity as 100 up to 3000 cpm and MVPA as more than 3000 cpm.

Children self‐reported sedentary behaviour using the *validated* Girls Health Enrichment Multi‐Studies physical activity survey 19. Children completed the survey with the assistance of a trained research assistant, and the meaning of each question was explained. Children were asked ‘Think about whether you usually watch TV, Videos/play computer games, video games/Homework, reading. Now please check the box that best fits how much time you spent doing each activity.’ Response options were as follows: none/less than 30 min/30 min–1 h/1–3 h/more than 3 h.

### Exposure measure: ethnicity

Children self‐reported their ethnicity (Caucasian, mixed, South Asian, Black). Ethnicity was then categorized as (Caucasian/mixed, Black, South Asian). Child self‐reported ethnicity has been successfully used in previous similar studies 15.

### Overweight/obesity

Trained research assistants measured participants' weight using the Tanita SC‐330 Body Composition Analyser (Tanita Inc, IL, USA) and height using the Leicester Height Measure, from which body mass index (BMI) was derived in kg m^−2^. Participants were then categorized as normal weight/overweight/obese using the BMI International Obesity TaskForce age/sex‐specific cut offs 20.

### Participant characteristics

A total of 450 participants from seven intervention and two control schools were recruited into the study at baseline. Valid baseline Actigraph data were provided in 396 children, and of those, 281 (71%) completed follow‐up. Reasons for drop‐out included left school/absent on day of follow‐up data collection (*n* = 46), insufficient wear time (*n* = 28) and failure to return device (*n* = 41). Participants that completed the baseline assessment but not follow‐up were slightly older (9.8 vs. 9.0 years, *p* = 0.01) than the children in the full analytic sample but no differences in sex distribution (49.6% vs. 47.7% boys) or ethnicity (23.5% vs. 18.0% South Asian in drop‐outs and final sample, respectively) were noted.

In the analytic sample, 64% were Caucasian/mixed, 18% Black and 18% South Asian. The descriptive characteristics are displayed in Table 1. Approximately 10% (*n* = 27) of the sample were categorized as obese (using BMI International Obesity TaskForce age/sex‐specific cut offs; Table 1). We found no statistical differences in obesity between ethnic groups (Chi‐square test, *p* = 0.77). At baseline, 77.6% of the sample recorded at least 4 days of Actigraph wear and 6.4% only 1 day of wear.

**Table 1:** Baseline characteristics of sample

	Caucasian/Mixed(*n* = 179)	Black(*n* = 51)	South Asian(*n* = 51)
Age	8.7 ± 2.0	9.0 ± 2.1	9.9 ± 1.9
Sex (% male)	50.8	47.0	37.3
BMI (kg m^−2^)	18.1 ± 3.8	18.2 ± 4.1	18.2 ± 4.0
*Weight Status* *			
Normal weight	126 (71.2)	37 (74)	40 (78.4)
Overweight	31 (17.5)	9 (18)	8 (15.7)
Obese	20 (11.3)	4 (8)	3 (5.9)
Total Actigraph wear time (min d^−1^)	741 ± 90	775 ± 83	777 ± 100
Total days wear	4.6 ± 1.7	4.7 ± 1.4	4.8 ± 1.8
MVPA (min d^−1^)	28 ± 15	32 ± 20	20 ± 12
Light PA (min d^−1^)	363 ± 74	375 ± 49	355 ± 84
Sedentary (min d^−1^)	349 ± 86	369 ± 81	403 ± 99

* Categorized using BMI International Obesity TaskForce age/sex‐specific cut offs.

BMI, body mass index; MVPA, moderate and vigorous physical activity; PA, physical activity.

### Analyses

Characteristics of the study population at baseline were analysed using descriptive statistics. Mixed models, adjusted for age, sex, the ratio of MVPA/wear time at baseline, intervention or control group (fixed effect) and school (random effect, to account for clustering at the school level) were employed to compare ratio MVPA/wear time at follow‐up between ethnic groups. We also examined change in sedentary time as the outcome, but for this analysis, we adjusted for the ratio of baseline sedentary/wear time instead. Analyses were performed using SPSS version 22 (Chicago, IL, USA), and statistical significance was set at *p* < 0.05.

## Results

At baseline, South Asians recorded less time in MVPA compared with Caucasians/mixed (B = −6.5, 95% confidence interval [CI], −11.1, −1.9 min d^−1^, *p* = 0.006) after adjusting for age, sex, wear time and school. There were no differences in MVPA between Black and Caucasian/mixed children (B = 3.4, 95% CI, −1.2, 8.0, *p* = 0.14). There were no differences in sedentary time between South Asians and Caucasians/mixed (B = 9.7, 95% CI, −7.5, 26.8 min d^−1^, *p* = 0.27) or Black and Caucasian/mixed children (B = −5.2, 95% CI, −22.1, 11.7 min d^−1^, *p* = 0.54) after covariate adjustments.

**Figure 1:**
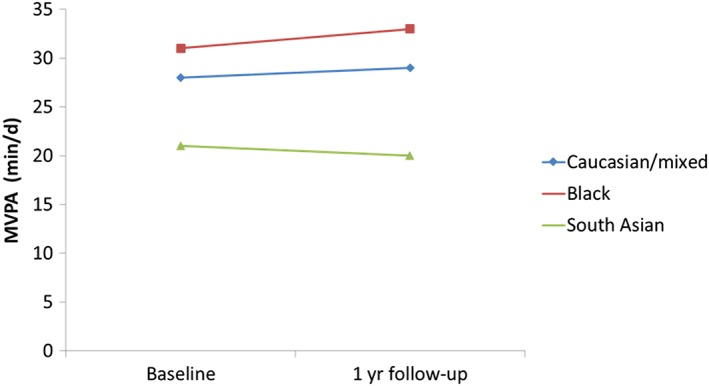
Changes in moderate and vigorous physical activity in relation to ethnicity (*n* = 281). [Colour figure can be viewed at wileyonlinelibrary.com]

**Figure 2:**
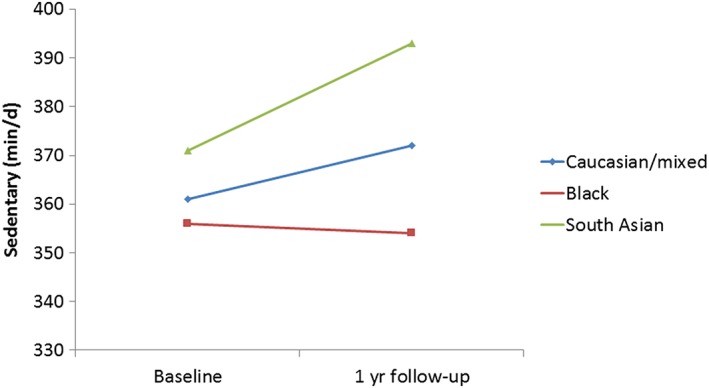
Changes in sedentary time in relation to ethnicity (*n* = 281). [Colour figure can be viewed at wileyonlinelibrary.com]

In adjusted mixed models to examine changes over time, there were no differences in MVPA between ethnic groups (Table 2; Fig. 1). South Asians, however, had a significantly greater increase in time spent sedentary at follow‐up than those of a Caucasian/mixed ethnicity (B = 0.024; 95% CI 0.003, 0.046) (Table 2, Fig. 2).

**Table 2:** Mixed models examining changes in physical activity and sedentary time in relation to ethnicity

Caucasian/Mixed	Coefficient (95% CI)[Link] for ratio MVPA/wear time at follow‐up	Coefficient (95% CI) for ratio sedentary/wear time at follow‐up
	Ref	Ref
Black	0.001 (−0.005, 0.007)	−0.019 (−0.040, 0.002)
South Asian	−0.006 (−0.013, 0.0005)	0.024 (0.003, 0.046)*

* *p* < 0.05.

^†^Coefficient adjusted for age, sex, and the ratio of MVPA or sedentary/wear time at baseline, intervention or control group (as fixed effects) and school (as random effect).

CI, confidence interval; MVPA, moderate and vigorous physical activity.

In further analyses, we incorporated participants' weight status (normal weight/overweight/obese; categorized using BMI International Obesity TaskForce age/sex‐specific cut offs) as a covariate; this made no substantial differences to the results (results not shown). We examined changes in self‐reported sedentary behaviours. At baseline, South Asian children tended to report greater TV viewing and computer usage, although at follow‐up, these differences were less apparent (Table 3). South Asian children reported greater homework, and this remained evident at follow‐up.

**Table 3:** Self‐reported sedentary behaviours at baseline and follow‐up

	Caucasian/Mixed		Black		South Asian	
	Baseline	Follow‐up	Baseline	Follow‐up	Baseline	Follow‐up
TV viewing (min d^−1^)						
<30	43.5	28.5	48.8	29.2	30.6	37.5
30 to 60	35.4	38.4	26.7	33.3	32.6	33.3
>60	21.1	32.0	24.4	37.5	36.7	29.2
Computer (min d^−1^)						
<30	56.8	46.0	62.2	42.9	47.9	56.3
30 to 60	24.7	29.9	11.1	30.6	25.0	22.9
>60	17.9	24.1	26.7	26.5	27.1	20.8
Homework (min d^−1^)						
<30	45.7	41.3	42.2	46.9	26.5	29.1
30 to 60	28.4	36.8	31.1	28.6	53.1	43.8
>60	25.9	21.8	26.7	24.5	19.6	27.1

Data presented as percentages.

## Discussion

Previous cross‐sectional studies of children have reported ethnic differences in physical activity. This is the first study to examine longitudinal changes in a multi‐ethnic sample of children. Our data show that children of South Asian ethnicity exhibited a significantly greater increase in sedentary time over a period of 12 months in comparison with Caucasian/mixed children. No significant differences were observed in 12‐month changes in MVPA between ethnic groups, although South Asian children displayed lower MVPA at baseline. These findings support and add to previous cross‐sectional research that shows children of a South Asian ethnicity residing in the UK have the lowest levels of activity 13, 14. This is of particular concern as South Asians are at a significantly increased risk of type II diabetes early in life 2, 3. Indeed, low levels of physical activity and high levels of sedentary behaviour are associated with incident diabetes 8, 9, 10, possibly through promoting visceral adiposity and impaired glucose control 21, 22. However, the aetiology of increased metabolic risk in South Asians is incompletely understood.

The self‐reported data on sedentary behaviours provided some context to help interpret the objective data and largely confirmed the generally higher prevalence of sedentary activities such as TV viewing, computer use and homework in the South Asian children. Nevertheless, increases in self‐reported sedentary behaviours over follow‐up were less marked than objectively measured sedentary time in South Asian children. This is perhaps unsurprising as many physical activity questionnaires, including ours, have not been validated to examine changes over time. In addition, self‐reported measures of sedentary behaviour are often poorly correlated with objective data from motion sensors 23.

The reasons for observed differences in activity behaviour across ethnic groups in the UK are poorly understood. However, given that similar trends have been reported in adult samples 24, it is unsurprising that these results are replicated in children because parental physical activity is a strong predictor of their children's activity 25. Socio‐cultural factors may play a key role 26, for example, some conventional physical activities may not fit within the health beliefs and culture of South Asian communities.

In a recent qualitative study in South Asian *adults*, a number of barriers to participation in physical activity were described and appropriate intervention content recommended 27. Key barriers included the following: issues with personal safety, cost, family and childcare commitments and the availability of free time. The study suggests that the use of the following will be relevant to intervention development: enhancing physical activity by increasing walking; use of pedometers; multilingual spoken content and delivery; peer support, for example, bilingual community members, to facilitate engagement and motivation; undertaking activity with families or friends; and delivery in local informal settings including the home. While some of these may be appropriate for South Asian children, they are adult‐specific. To the best of our knowledge, similar data for South Asian children residing in the UK do not exist.

A clear strength of the present study is the longitudinal design in an ethnically diverse sample of children residing in London, UK. We adjusted for school as a random effect, to account for clustering at the school level, thus results are unlikely to have been explained by local area factors. The 1‐year interval separating baseline and follow‐up data collection ensured seasonally matched data, thereby reducing the risk of artefactual changes in activity attributable to different weather conditions at two time points. We chose to use a conservative cut point to derive MVPA; thus, some moderate intensity activity could have been misclassified as light activity. Nevertheless, the relatively low levels of physical activity in the present study are consistent with data from a prior survey using self‐report where only 12% of children from Camden met the physical activity guideline 28; www.camden.gov.uk/jsna.

As in previous studies 13, registered wear time differed between ethnic groups, with Caucasians/mixed recording the lowest average wear period per day. Although analyses adjust for wear time, it is unknown what activities the children undertake during non‐registered wear. However, if Caucasian/mixed children were undertaking physical activity (such as water sports) during non‐registered wear periods, the ethnic differences observed may reflect a conservative estimate of the true differences. Data on individual South Asian groups (e.g. Indian/Pakistan/Bangladesh) were not collected, and thus, it was not possible to investigate differences in activity levels between groups. However, a recent study found that levels of estimated VO_2max_ were lower in South Asian children than those in white Europeans and the lower estimated VO_2max_ in South Asians, compared with white Europeans, was consistent among Indian, Pakistani and Bangladeshi children. This suggests that any differences in physical activity levels between South Asian groups are likely to be minimal 29. Given the age range of the children in the present study, some children may have been entering puberty at the time of data collection, which could potentially confound the results presented. However, data on stage of maturation were not collected. Future studies should control for such data in analyses.

## Conclusion

In this ethnically diverse sample of children, those of a South Asian ethnicity exhibited a significantly greater increase in sedentary time over a period of 12 months in comparison with Caucasian/mixed children. Sedentary habits established in early life may have implications for diabetes, particularly in high‐risk groups such as South Asians.

## Conflict of Interest Statement

No conflict of interest was declared.

## Funding source

This work was supported by The Economic and Social Research Council, UK (ES/M003795/1), and London Borough of Camden. The funders had no role in the study design; in the collection, analysis and interpretation of data; in writing of the report; or in the decision to submit the paper for publication.

## Contribution Statement

L. S. conceptualized and designed the study, drafted the manuscript and approved the final version. D. A. designed the study, collected the data and approved the final manuscript. M. H. conceptualized and designed the study, drafted the manuscript and approved the final version.
